# Public Awareness about Antibiotic Use and Resistance among Residents in Highland Areas of Vietnam

**DOI:** 10.1155/2019/9398536

**Published:** 2019-05-16

**Authors:** Thuy Van Ha, An Mai Thi Nguyen, Ha Song Thi Nguyen

**Affiliations:** ^1^Department of Health Insurance, Ministry of Health of Vietnam, Hanoi 100000, Vietnam; ^2^Department of Planning and Financing, Ministry of Health of Vietnam, Hanoi 100000, Vietnam; ^3^Hanoi University of Pharmacy, Hanoi 100000, Vietnam

## Abstract

**Background:**

Antibiotic resistance (AR) remains a global crisis. However, the literature on public awareness about antibiotic use and AR in the highland provinces of Vietnam has been constrained. This study explores the awareness of antibiotic use and resistance among general people in highland provinces in Vietnam and detects associated factors.

**Methods:**

A cross-sectional study was performed in five highland provinces with 1000 households. Information about socioeconomic status and awareness regarding prescription medicine use, antibiotic use, and AR was surveyed. Multivariate logistic regression was used to identify associated factors with awareness.

**Results:**

64.2% of people were aware of prescription drugs. More than two-thirds (67.4%) of participants were aware of antibiotic use, of whom only 55.8% were aware of AR. Higher age, education, and family income were positively associated with being aware of prescription medicine, antibiotic, and AR. Females had a lower likelihood of being aware of prescription medicine (OR=0.64; 95%CI=0.45-0.90) compared to male counterparts. Those being freelancers were more likely to be aware of antibiotic resistance (OR=2.30; 95%CI=1.13-4.67) compared to those working in agriculture/fishery/forestry sector. Compared to Kinh ethnic, most ethnic minorities were less likely to be aware of prescription medicine, antibiotic, and AR.

**Conclusions:**

This study showed a low awareness regarding prescription medicine, antibiotic use, and AR among public people in the highland provinces of Vietnam. Further systemic and didactic educational interventions targeting females, low education, low income, ethnic minorities, and those working in agriculture/fishery/forestry sector in this setting should be performed and evaluated to improve the awareness about antibiotic use and resistance.

## 1. Introduction

Antibiotic resistance (AR) is a condition when bacteria change against antibiotics which are developed to cure the illnesses they cause [1]. It is a global crisis and posed as one of the greatest threats to population health. This problem is driven by many factors such as low quality of antibiotics and improper (under- or over-) use of antibiotics (including self-medication) [1–3]. Self-medication, which refers to the use of any medical products without a prescription or following unprofessional recommendations in treating any illnesses [4, 5], is particularly leading to the AR. Self-medication practice possibly raises incorrect drug selection, drug resistance, uncontrolled adverse effects or drug reactions, misdiagnosis, and delay in medical care [6–8]. Self-medication is a common phenomenon, and the prevalence varies from 12.7% to 18% in Spain [9, 10], 32% to 45.4% in China [11, 12], 53% in Mexico [13], and 75% in the United Kingdom and Chile [14, 15]. Also, the excessive antibiotic utilization in the agriculture sector causes the pool of AR bacteria in the animals, which are then transferred to the human through consuming food from these animals [16, 17]. It is estimated that, in 2050, there will be more than 10 million deaths and 100 trillion USD lost due to AR if no substantial actions have been made to eliminate this emerging threat [18–20].

As inappropriate antibiotic use is the primary cause of AR, responses to this phenomenon prioritize to promote public awareness about AR [21]. Nonetheless, it is evidenced that public awareness about AR is insufficient even in wealthy countries [22, 23], and it is more severe in low- and middle-income countries, where antibiotic use without a prescription is prevalent [24–26]. For example, a recent survey conducted by the World Health Organization indicated that most of the respondents in developing countries believed that antibiotics could be used to treat viral infections [21]. Many educational interventions have been conducted worldwide to enhance awareness, knowledge, and practice in antibiotic use; however, the effects were not clear and varied across study settings [27–29]. Thus, further evidence on public awareness about AR in specific settings should be required, which can be used to contextualize and optimize the effectiveness of interventions.

In Vietnam, the inappropriate use of antibiotics and prescription drugs are pervasive, particularly in hospitals. A study in Ho Chi Minh City found that 93% of patients admitted to the intensive care unit had resisted to at least one antibiotic [30]. Another study showed that about one-third of patients experienced misuse of prescription drugs [31]. Nguyen et al. indicated that 63% of children in rural areas used unnecessary antibiotics, which most of them were prescribed by health professionals, to treat acute respiratory infections [32]. Moreover, Do et al. found that 88% and 91% of antibiotics in urban and rural were sold without a prescription, respectively [33]. However, the literature on public awareness about AR in highland provinces, which had a diversity of ethnicities with different cultural approaches in treating diseases, has been constrained. This study, therefore, aims to explore the awareness of antibiotics use and AR among general people in the highland provinces of Vietnam and detect potential associated factors. This study could be served as a baseline survey for further interventions to promote the proper use of antibiotics in this population.

## 2. Materials and Methods

### 2.1. Study Designs and Sampling Method

We conducted a cross-sectional survey from July to November 2018 in five highland provinces of Vietnam including Kon Tum, Gia Lai, Dak Lak, Dak Nong, and Lam Dong. A multistage sampling method was employed to recruit participants. First, a list of districts and communes in each province was developed by the local authorities. A total of 721 communes in 62 districts were listed in the sample frame. Then, by using computer software, we randomly selected two districts in each province (10 districts/5 provinces), and after that, two communes per district (20 communes/10 districts). Then, we randomly selected five villages in each commune and ten households per village. Door-to-door recruitment strategy was used to enroll participants until reaching sufficient sample size.

The sample size was calculated by using the formula for estimating a population proportion with specified absolute precision. We expected that the prevalence of residents being aware of AR was 50%. With a marginal error (absolute precision) = 0.07 and a confident level = 95%, the total sample size for each province was 196 households. We also added 5% of sample size for preventing if the household's representative refused to participate or they incompletely answered the interview. A total of 1029 households were randomly selected from the sampling frame. The participants were invited if they were 1, aged from 18 years or more, and 2, head of households or individuals who were responsible for purchasing medicines for the household.

### 2.2. Data Collection and Measurements

Face-to-face interviews were performed to collect the data from 15 to 20 minutes. A structured questionnaire was developed for the survey. A pilot survey was conducted with ten households in Dak Lak province. Data of these households were not included in the final dataset. After the pilot, we received the feedbacks from households about the content, language, and logical order of items in the questionnaire. A major revision was performed to ensure the cultural appropriateness of the questionnaire. The final version of questionnaire was produced and approved by the local authorities. A two-day training course was conducted by the principal investigator and the research team who had expertise in antibiotic use and resistance. Interviewers consisted of local health workers who had experiences in working with households. They were informed about the objectives of the survey and the content of the questionnaires, as well as trained necessary interview skills to collect data consistently with high quality.

The questionnaire included information about the socioeconomic status of respondent (age, gender, ethnic, education, marital status, and occupation) and household (number of family members, number of children in the family, number of people having health insurance in the family, and annual household income). People belonging to other ethnics instead of “Kinh” ethnic were classified as “ethnic minorities” since “Kinh” has been the dominant ethnic in Vietnam.

The other parts of the questionnaire were questions about “awareness of prescription drug”, “awareness of antibiotic use”, and “awareness of antibiotic resistance”. In this study, antibiotics are defined as drugs that are used to “prevent and treat bacterial infection” according to the World Health Organization [1]. Our questions were referred from previous studies in the world. Respondents were asked to report whether they were aware of prescription drugs by defining prescription drugs accurately [34]. We also asked patients to respond whether they were aware of antibiotic and AR (including definition of AR) (“Have you ever heard of antibiotics/antibiotic resistance?” – Yes/No; “If you have heard, do you know what antibiotic resistance means?”) [35, 36], whether they knew that using antibiotic required prescriptions (“Do you need a prescription when you use antibiotics?” – Yes/No/Don't know) [37], who qualified prescribers were (“If you have to use antibiotics, whose prescription should you follow?”) [35, 36], solutions when health was not improved after the course of antibiotic (“If your health is not improved after the antibiotic course, what will you do?”) [38], and negative effects of antibiotic use (“In your opinion, what are the negative effects of antibiotics use?”) [38, 39]. Details of the questionnaire were informed in the Supplementary File 1.

### 2.3. Statistical Analysis

Data were analyzed using Stata version 14.0. Descriptive analysis including mean, standard deviation, frequency, and the percentage was performed to describe the variables. Two-tailed chi-squared and student t-tests were used to assess the differences in socioeconomic characteristics and awareness between males and females. Multivariate logistic regression was used to identify the factors associated with “Being aware of prescription drugs” and “Being aware of antibiotic”, and “Being aware of AR”. The independent variables included in the models were socioeconomic status of respondent (age, gender, ethnic, education, marital status, and occupation) and household (number of family members, number of children in the family, number of people having health insurance in the family, and household income quintiles). Stepwise backward selection strategies were applied in combination with regression models, using a p-value of log likelihood test at 0.2 as a threshold for selecting the variables to the reduced models. A p-value of less than 0.05 was treated as statistical significance.

### 2.4. Ethical Consideration

The study protocol was approved by the Institutional Review Board of the Vietnam Ministry of Health. Participants had the rights to stop the interview or withdraw to the study at any time without any barriers. Written informed consent was obtained if they agreed to participate in the study. They did not receive any incentives or reimbursement for participation. The questions were anonymous, and no personal information was collected during the interview.

## 3. Results

Among 1029 households selected, after excluding households that had incomplete data, information of 1000 households was used for data analysis (response rate 97.2%). A total of 1000 people in 1000 households participated in the study. The demographic characteristics of respondents are indicated in Table 1.

There were 64.2% of people being aware of prescription drugs. This rate in males was significantly higher than that in females (p<0.05). More than two-thirds (67.4%) of participants were aware of antibiotics. Most of them understood that using antibiotic required prescriptions (83.5%), of whom 94.9% were aware that antibiotics had to be prescribed by physicians at the medical facility. There were 8.0% concerning drug sellers at the drug store as qualified prescribers. More than half of participants (53.9%) would reexamine health if antibiotics worked ineffectively, while above one-fifth of people (21.8%) would replace to other antibiotics. Only 18.8% knew AR as a negative effect of antibiotic use, and only 55.8% were aware of AR. Most people being aware of AR knew that AR was where bacteria changed to respond to the use of antibiotic, while only 25.3% knew the causes of AR. (Table 2)

Higher age, education, and family income were positively associated with being aware of prescription medicine, antibiotics, and AR. Females had a lower likelihood of being aware of prescription medicine (OR=0.64; 95%CI=0.45-0.90) compared to males. Those being freelancers were more likely to be aware of antibiotic resistance (OR=2.30; 95%CI=1.13-4.67) compared to those working in agriculture/fishery/forestry sector. Compared to Kinh ethnic, most of the ethnic minorities were less likely to be aware of prescription medicine, antibiotic, and AR. People who were Mnong ethnic were more likely to know prescription medicine but less likely to know AR (Table 3).

## 4. Discussion

In this study, the results indicated a lack of awareness about appropriate prescription medicine use and antibiotic use as well as AR among the general population in highland areas of Vietnam. Moreover, by using multivariate regression model, we highlighted the most vulnerable populations to the insufficient awareness of these terms, including females, low age, low education, low income, ethnic minorities, and those working in agriculture/fishery/forestry sector.

In recent years, Vietnam has been a hotspot of AR in the world due to the shortage of legislation and regulations to manage self-medication as well as poor prescription drug and antibiotic use practices [40]. A previous study revealed that 83% of* Escherichia coli* found among wastewater samples in Vietnamese hospitals resisted to at least one antibiotic, and 32% could resist multiple antibiotics [41]. Despite the severity of this phenomenon, our findings revealed a limited awareness of highland people about antibiotic use and AR. For instance, only 64.2%; 67.4%, and 55.8% were aware of prescription medicine, antibiotic, and AR, respectively. Moreover, the result showed the superficiality in the knowledge of these groups since only one-fourth of individuals knowing AR could define the cause of AR. These proportions in our study were comparable to other countries. A study in Hong Kong found that 91% adults knew the term “antibiotic resistance” [42], and 59.0% Malaysians perceived that antibiotic misuse could lead to AR [43]. The results in different studies varied due to the different measures, settings as well as sociocultural-economic backgrounds. Nonetheless, the emerging situation of constrained awareness of population in highland provinces of Vietnam should be underlined; thus, further interventions should be elucidated to improve this issue.

Our study was in line with previous surveys worldwide that age, education, and gender were significant predictors to the awareness of antibiotics use [44–47]. Specifically, lower age, lower education, and females were less likely to be aware of prescription medicine, antibiotics use, and AR. A study in Poland found that people who were young and had lower education were more likely to believe that antibiotics could protect against viral infections [44]. Moreover, younger people had a higher likelihood of self-medication [48]. Notably, we found that those working in the agriculture /fishery/forestry sector had a lower chance of being aware of AR. It should be noted that food animal production is a potential source of AR due to the overuse of antibiotic during farming activities [49].

Meanwhile, the findings demonstrated the susceptibility of ethnic minorities in AR since they were less likely to be aware of antibiotic and AR. Ethnics minorities in Vietnam have been found to have limited health care service and health information access [50, 51]. Therefore, mandatory educational training about prescription medicine, antibiotic use, and AR should be reinforced to these populations to ensure sufficient knowledge about these health problems.

This study has several methodological issues that should be acknowledged. First, the causal relationships between the awareness and its associated factors could not be established due to the nature of study design. Second, the self-reported approach might result in recall bias when we asked them to report annual household income and social desirability bias when respondents might give the answers that were viewed favorably by interviewers. However, we have trained the data collection team carefully and developed the data collect guideline to reduce the bias. Third, several critical information was not included in the study, for example, participants' behaviors, impacts of family and social relationships, or health service quality. Moreover, although we conducted the study on a large scale with 1000 households, our generalizability might be limited and not represent to other settings. Thus, our findings should be used in caution. Further studies should be warranted to fill the knowledge gap in the awareness about prescription medicine use, antibiotic use, and AR among highland residents.

## 5. Conclusion

This study shows a low awareness regarding prescription medicine and antibiotic use among public people in highland provinces in Vietnam. The findings also indicated that females, low education, low income, ethnic minorities, and those working in agriculture/fishery/forestry sector should be the target groups in future interventions in this setting.

## Figures and Tables

**Table 1:** Sociodemographic characteristics.

Characteristics	Male		Female		Total		p-value
	n	%	n	%	n	%	
Total	421	42.1	579	57.9	1000	100.0	
Age group							
≤ 30 years old	153	36.3	238	41.1	391	39.1	<0.01
31-40 years old	165	39.2	139	24	304	30.4	
41-50 years old	46	10.9	91	15.7	137	13.7	
51-60 years old	38	9	75	13	113	11.3	
> 60 years old	19	4.5	36	6.2	55	5.5	
Occupation							
Work in agriculture / fishery / forestry sector	312	74.1	452	78.1	764	76.4	<0.01
White-collar worker	17	4	24	4.1	41	4.1	
Business	23	5.5	32	5.5	55	5.5	
Freelancer	44	10.5	9	1.6	53	5.3	
Retirement	7	1.7	16	2.8	23	2.3	
Housemaker	4	1	41	7.1	45	4.5	
Other	14	3.3	5	0.9	19	1.9	
Education							
Illiterate	28	6.7	93	16.1	121	12.1	<0.01
Elementary school	94	22.3	135	23.3	229	22.9	
Secondary school	156	37.1	211	36.4	367	36.7	
High school	104	24.7	94	16.2	198	19.8	
Vocational training/College	19	4.5	39	6.7	58	5.8	
University/Postgraduate	20	4.8	7	1.2	27	2.7	
Ethnic							
Kinh	239	56.8	211	36.4	450	45	<0.01
Gia rai	32	7.6	92	15.9	124	12.4	
E de	6	1.4	38	6.6	44	4.4	
Xo dang	29	6.9	42	7.3	71	7.1	
Mnong	47	11.2	55	9.5	102	10.2	
Cil	11	2.6	50	8.6	61	6.1	
Nung	17	4	16	2.8	33	3.3	
Ro ngao	18	4.3	47	8.1	65	6.5	
Other	22	5.2	28	4.8	50	5	
Household economic classification							
Poor	66	15.7	113	19.5	179	17.9	0.10
Near-poor	44	10.5	75	13	119	11.9	
Non-poor	311	73.9	391	67.5	702	70.2	

	Mean	SD	Mean	SD	Mean	SD	

Age	36.0	11.5	37.3	13.4	36.7	12.7	0.51
Number of members in family	5.0	1.8	4.8	1.8	4.9	1.8	0.11
Annual household income	82.8	73.3	76.1	118.8	79.0	102.1	<0.01

**Table 2:** Awareness and practice about safe antibiotic use.

Characteristics	Male		Female		Total		p-value
	n	%	n	%	n	%	
*Being aware of prescription drugs (n=1000)*	307	72.9	335	57.9	642	64.2	<0.01
*Being aware of antibiotic (n=1000)*	306	72.7	368	63.6	674	67.4	<0.01
*Need prescriptions when using antibiotic (n=674)*	266	86.9	297	80.7	563	83.5	0.09
*Prescribers (n=563)*							
Physicians at medical facility	246	92.5	288	97.0	534	94.9	0.02
Drug sellers at drug store	25	9.4	20	6.7	45	8.0	0.24
Friends/relatives/neighbourhoods	0	0.0	2	0.7	2	0.4	0.18
Old prescriptions	1	0.4	1	0.3	2	0.4	0.94
Other	3	1.1	0	0.0	3	0.5	0.07
*Solutions when health was not improved after the antibiotic course (n=674)*							
Replace to other antibiotics	62	20.3	85	23.1	147	21.8	0.38
Increase dose	7	2.3	12	3.3	19	2.8	0.45
Re-examination	175	57.2	188	51.2	363	53.9	0.11
Ask advices from drug sellers/physicians	59	19.3	121	32.9	180	26.7	<0.01
Ask friends/relatives/neighbourhoods	0	0.0	3	0.8	3	0.5	0.11
Other	19	6.2	15	4.1	34	5.0	0.21
Unknown	25	8.2	17	4.6	42	6.2	0.06
*Negative effects of antibiotic use (n=674)*							
AR	51	16.7	76	20.7	127	18.8	0.19
Drug allergy	86	28.1	105	28.5	191	28.3	0.90
Harm to health	125	40.9	151	41.0	276	41.0	0.96
Other	5	1.6	15	4.1	20	3.0	0.06
Unknown	77	25.2	92	25.0	169	25.1	0.96
*Being aware of AR (n=674)*	182	59.5	194	52.7	376	55.8	0.08
*Definition of AR (n=376)*							
Condition that bacteria change to respond the use of antibiotic	153	84.1	153	78.9	306	81.4	0.20
Occur when using antibiotics without prescriptions, or sufficient dosage or time	48	26.4	47	24.2	95	25.3	0.63
Other	5	2.8	10	5.2	15	4.0	0.23
Unknown	11	6.0	11	5.7	22	5.9	0.88

**Table 3:** Associated factors with the awareness about safe prescription medicine and antibiotic use.

Characteristics	Being aware of prescription medicine		Being aware of antibiotic		Being aware of AR	
	OR	95%CI	OR	95%CI	OR	95%CI
*Age*	1.03*∗∗*	1.02; 1.05			1.02*∗*	1.00; 1.03
*Education*	2.10*∗∗*	1.74; 2.54	2.08*∗∗*	1.76; 2.46	2.44*∗∗*	2.02; 2.94
*Gender*						
Male	ref					
Female	0.64*∗*	0.45; 0.90				
*Ethnic*						
Kinh	ref		ref		ref	
Gia rai	0.32*∗∗*	0.18; 0.56	0.18*∗∗*	0.10; 0.32	0.12*∗∗*	0.05; 0.30
E de	0.27*∗∗*	0.12; 0.60			0.32*∗*	0.13; 0.81
Xo dang	0.62	0.33; 1.17	0.41*∗∗*	0.22; 0.78	0.22*∗∗*	0.09; 0.57
Mnong	2.79*∗∗*	1.54; 5.05	1.60	0.96; 2.66	0.50*∗*	0.27; 0.93
Cil					0.65	0.34; 1.24
Ro ngao	0.71	0.35; 1.43	0.26*∗∗*	0.12; 0.56	0.32*∗*	0.11; 0.94
*Occupation*						
Work in agriculture/fishery /forestry sector	ref		ref			
Freelancer			1.86	0.82; 4.20	2.30*∗*	1.13; 4.67
Retirement	5.60	0.69; 45.37				
*Household income quintile*						
Poorest	ref		ref		ref	
Middle	2.00*∗∗*	1.28; 3.11	1.62*∗*	1.07; 2.47	1.90*∗∗*	1.17; 3.08
Rich	2.88*∗∗*	1.74; 4.75	2.55*∗∗*	1.62; 4.02	2.56*∗∗*	1.59; 4.13
Richest	4.83*∗∗*	2.59; 9.00	4.07*∗∗*	2.38; 6.95	4.20*∗∗*	2.48; 7.10
*Number of members in family*	0.92	0.84; 1.01	0.92	0.84; 1.00		

*∗∗*p<0.01; *∗*p<0.05.

## Data Availability

Data in this manuscript belongs to the Vietnam Ministry of Health. Please contact the corresponding author for further request.

## Supplementary Materials

Supplementary Materials
* Supplementary File 1: questionnaire of this study*. This questionnaire included information collected in the study.
