# Supplementary material for: Public Awareness about Antibiotic Use and Resistance among Residents in Highland Areas of Vietnam
Source: Biomed Res Int. 2019 May 16;2019:9398536. doi: 10.1155/2019/9398536 (PMC6541961; doi:10.1155/2019/9398536)
Supplement: Supplementary Materials — Supplementary File 1: questionnaire of this study. This questionnaire included information collected in the study. [file 9398536.f1.docx]

**QUESTIONNAIRE**

| **STT** | **CÂU HỎI** | **TRẢ LỜI** | |
| --- | --- | --- | --- |
|  | Age |  | |
|  | Gender | 1. Male 2. Female | |
|  | What is your ethnicity? | 1. Kinh 2. H’Mông 3. Gia rai 4. Xtiêng 5. Chu ru 6. Mường 7. Ê đê 8. Xơ đăng 9. Mnông | 1. Hrê 2. Ba na 3. Cơ ho 4. Giẻ triêng 5. Ra Glai 6. Mạ 7. Thái 8. Other (specify:........................) |
|  | What is your education level? | - 1. Illiterate   2. Elementary   3. Secondary   4. High school   5. Vocational training/College   6. University/postgraduate | |
|  | What is your current main occupation? | 1. Work in agriculture / fishery / forestry sector 2. White-collar worker 3. Business 4. Freelancer 5. Retirement 6. Housemaker 7. Other (specify…………………………….) | |
|  | How many people living in your household? | *people*  *Number of children under 5 years old: ……………* | |
|  | How many people in your household had health insurance card? | *people* | |
|  | What is your household’s annual income? | .................................VND | |
|  | What is your household’ economic classification according to the government standard? | - 1. Poor   2. Near-poor   3. Non-poor | |
|  | Do you know what prescription drugs are?  *(Multiple choices)* | 1. Prescription drugs are medicines that must be prescribed by the prescriber / physician when used 2. Prescription drugs are drugs that, if used not according to physician' prescriptions, may be dangerous to life and affect health negatively. 3. Other (specify) ........................................... ................... 4. Don't know | |
|  | Have you ever heard of antibiotics? | - 1. Yes   2. No | |
|  | Do you need a prescription when you use antibiotics? | 1. Yes 2. No 3. Don’t know | |
|  | If you have to use antibiotics, whose prescription should you follow? | 1. Physicians at medical facility 2. Drug sellers at drug store 3. Friends/relatives/neighbourhoods 4. Old prescriptions 5. Self-experience 6. Other (specify:…………………………………………….) | |
|  | If your health is not improved after antibiotic course, what will you do? | 1. Replace to other antibiotics 2. Increase dose 3. Re-examination 4. Ask advices from drug sellers/ physcians 5. Ask friends/relatives/neighbourhoods 6. Other (specify:…………………………………………….) 7. Unknown | |
|  | In your opinion, what are the negative effects of antibiotics use? | 1. Antibiotic resistance 2. Drug allergy 3. Costly 4. Harm to health 5. Other (specify: ………………………………………………….) 6. Unknown | |
|  | Have you ever heard of antibiotic resistance? | 1. Yes  2. No | |
|  | If you have heard, do you know what antibiotic resistance means? | - 1. Condition that bateria change to respond the use of antibiotic   2. Occur when using antibiotics without prescriptions, or sufficient dosage or time   3. Other (specify: ………………………………………………….)   4. Unknown | |
